# Supplementary material for: Experimental study on tailings cementation by MICP technique with immersion curing
Source: PLoS One. 2022 Aug 1;17(8):e0272281. doi: 10.1371/journal.pone.0272281 (PMC9342764; doi:10.1371/journal.pone.0272281)
Supplement: Attachment — Submitted filename: Response to Reviewers.docx [file pone.0272281.s002.docx]

Dear Editor and Reviewers,

Thank you very much for giving us an opportunity to revise our manuscript. We appreciate very much for your positive and constructive comments and suggestions on our manuscript entitled " Experimental study on tailings cementation by MICP technique with immersion curing".

We have revised the manuscript in accordance with the reviewers’ comments, and carefully proofread it to avoid inaccurate expression and minimize similar errors that may arise. Below are our responses to the comments.

**Responses to Reviewers’ Comments**

The modifications indicated below are marked in red in the “Revised Manuscript with Track Changes”.

**Reviewer #1:**

**Comment**

1. Line 15: what does “ore energy” mean? Does it mean ore and energy?
**Response**

Thanks for the comments. The description here should be ore. We have revised the manuscript accordingly.

In addition, production of Portland cement results in consumption of a great amount of ore ~~energy~~ and air pollution.

**Comment**

2. Line 36: There should be always a space between worlds and bracket. e.g. should be “underground mining (Li et al. 2021; Wang et al. 2018)” instead of “underground mining(Li et al. 2021; Wang et al. 2018)”.

**Response**

Thank for the suggestion. There is indeed a lack of spaces in the text, and the reference format does not meet the journal requirements. The references in the revised manuscript have been modified to the appropriate format.

The shallow resources are gradually exhausted, and open-pit mining gradually transits to underground mining [1,2].

**Comment**

3. Lines 45-47: the expression sounds a bit weird. It should be rephrased.

**Response**

Thanks for the comment. We have made changes to follow the journal format.

Therefore, finding a tailings cementing material that can replace Portland cement has become a hot scientific research direction. The use of environmentally friendly tailings cementitious materials is of great significance for reducing carbon dioxide emissions and achieving carbon neutrality as soon as possible.

**Comment**

4. Lines 54-57: The description of urea hydrolysis is improper. Urea hydrolysis actually refer to the reaction process, in which urea is consumed, and carbonate ion and NH_4_ are produced. The authors should also mention that in the MICP treatment expect for urea and bacteria, Ca^2+^ should be additionally provided in the system. For better explanation of the chemical reactions by MICP, one should give the chemical equation here.

**Response**

Thanks for the suggestion. This problem has been explained in detail in the manuscript.

Urea hydrolysis refers to the continuous production of highly active urease by specific microorganisms (urease bacteria) in the process of metabolism. Urease will promote the hydrolysis of urea to generate carbon dioxide and ammonia, which combine with water to generate carbonate ions and ammonium ions, and increase the alkalinity of the environment. When calcium ions are added externally, calcium carbonate precipitates are formed in the environment. The reaction equation is shown in (1-6). ~~The alkalinity of the environment is enhanced due to carbon dioxide formed by urea hydrolysis, promoting the precipitation of Ca~~^~~2+~~^ ~~in the environment as calcium carbonate crystals.~~ The mechanism of the MICP technique based on urea hydrolysis is relatively simple and a large amount of calcium carbonate is precipitated within a short period. It is highly efficient, sustainable and pollution-free, and hence has attracted attention from many researchers around the world.

$CO\left(NH_{2}\right)_{2}+H_{2}O\to CO_{2}+2NH_{3}$ (1)

$CO_{2}+H_{2}O\leftrightarrow H_{2}CO_{3}$ (2)

$2NH_{3}+2H_{2}O\leftrightarrow2NH_{4}^{+}+2OH^{-}$ (3)

$2OH^{-}+H_{2}CO_{3}\leftrightarrow CO_{3}^{2-}+2H_{2}O$ (4)

$Ca^{2+}+Call\to Call-Ca^{2+}$ (5)

$Call-Ca^{2+}+CO_{3}^{2-}\to CaCO_{3}\downarrow$ (6)

**Comment**

5. Section 4.1: Any explanation for the experimental observation that after excess 1.6 OD, no significant increase of calcite production was observed? Actually this observation is quite common. With the increase of bacterial amount, the urease activity is improved. However, after reaching a certain bacterial mass competition among the bacteria increases due to the insufficient nutrients which on the contrary leads to the increase of bacterial decay thus decreases the activity. This can usually be seen in the growth rate of biomass. By means of changing the ambient environment of bacteria e.g. changing the nutrition receipts, or providing more nutrition) the bacteria activity can be further improved. Considering this, it is more interesting to investigate the optimal ratio of the bacterial concentration with respect to the urea/calcium concentration rather than to study these two concentrations separately.

**Response**

Thanks for the comment. In this paper, it is found through experiments that when the concentration of the cementing solution is constant, with the increase of the concentration of the bacterial solution, the cementing effect of the tailings shows a trend of first rising and then flattening. When the concentration of the bacterial solution is constant, with the increase of the concentration of the cementing solution, the cementing effect of the tailings first increases and then decreases. The single control variable method was adopted in this research process, and the optimal proportional relationship between the concentration of bacterial solution and the concentration of cementation solution was not considered. However, through this study, we can also see the change rule of the cementation effect of microorganisms on tailings under the influence of the concentration of bacteria solution or cementation solution alone.

**Comment**

6. Section 4.2: not sure if the conclusions drawing here make sense. Firstly, this observation is of no general applicability. Second, if one want to establish the relationship between urea concentration and bacterial urease activity, it is better to analyze the urease activity of bacteria in cases of different combination of bacteria concentration and urea concentration based on monitoring the decrease rate of urea mass during the test (e.g see. Xiao et al., 2020).

**Response**

Thanks for the comment. The purpose of this paper is to explore the influence of some influencing factors on the cementation effect in the process of microbial cement the tailings from a macroscopic point of view. Because the amount of calcium carbonate generated directly affects the strength of the specimen, this study mainly characterized the amount of calcium carbonate precipitation induced by microorganisms by testing the mechanical strength of the tailings specimen. Similar research methods can also be seen in the following articles. (Deng X, Yuan Z, Li Y, et al (2020) Experimental study on the mechanical properties of microbial mixed backfill. Constr Build Mater 265:. https://doi.org/10.1016/j.conbuildmat.2020.120643) The reviewer's analysis of bacterial urease activity under different combinations of bacterial concentration and urea concentration is a good research direction, and we will consider adopting it in subsequent research.

**Comment**

7. Section 4.3: Only uniform and homogenous distribution of different particle sizes is investigated. However, in the in-situ MICP cementation of tailing, the heterogeneous particle size distribution in a large ´field is expected.

**Response**

Thanks for the comment. The tailings used in this study are only the tailings collected in-situ in a tailings pond. When studying the effect of particle size on the cementation effect of tailings, the tailings with different particle sizes were ground based on the tailings in the tailings pond. When the technology is mature, when cementing the tailings in a specific tailings pond, it is necessary to analyze the particle size of the tailings, and select appropriate experimental parameters according to different particle size gradations.

**Comment**

8. Section 4.4: To analyze the relationship between temperature and spatial distribution of produced calcite, one should look into the urease activity of bacteria at different temperature (see e.g. Xiao et al., 2020).

Reference:

Xiao, Y., Wang, Y., Wang, S., Evans, T. M., Stuedlein, A. W., Chu, J., … Liu, H. (2021). Homogeneity and mechanical behaviors of sands improved by a temperature-controlled one-phase MICP method. Acta Geotechnica, 4.

**Response**

Thanks for the comment. This comment is very professional and visionary. The urease activity of bacteria will directly affect the progress of urea hydrolysis. There are many previous studies on the effect of bacterial urease activity. We believe that this paper mainly studies the effect of temperature and other conditions on the effect of solidification tailings by MICP technology from a macro perspective. This paper mainly studies the solidification effect of facultative anaerobic bacteria and aerobic bacteria on tailings, and the urease activity of bacteria has no effect on the denitrification of facultative anaerobic bacteria, so there is no study on the effect of temperature on bacterial urease activity Impact.

**Reviewer #2:**

**Comment**

1. The authors should be careful when writing the scientific names of bacteria species. The species name should be in italic - revise throughout the manuscript.

**Response**

Thanks for the comment. This problem has been explained in detail in the manuscript.

In this paper, a tailings cementation method by using the microbial induced calcite precipitation (MICP) technique with immersion curing is proposed. Tailings are cemented by the MICP technique with aerobic bacteria (*Sporosarcina pasteurii*) under a soaked curing environment. The variable control method is applied to investigate the factors influencing the cementation effects by the MICP technique with *Sporosarcina pasteurii*, including the bacterial solution concentration, the cementing solution concentration, the particle size of tailings, and the curing temperature. The results indicate that: when OD_600_ of the *Sporosarcina pasteurii* solution is 1.6, the urea concentration in the cementing solution is 0.75mol/L, the tailings are raw materials without grinding, and the curing temperature is 30℃, the cementation effect is the best. In view of uneven calcification during MICP with *Sporosarcina pasteurii*, mixed *Sporosarcina pasteurii* and *Castellaniella denitrificans* are used for tailings cementation. Higher strength of cemented tailings is achieved. It is proved that the MICP technique with mixed aerobic bacteria and facultative anaerobes is an effective method for tailings cementation.

To sum up, a tailings cementation method by mixed facultative anaerobes (*Castellaniella denitrificans* or *C. denitrificans* ) and *Sporosarcina pasteurii* (*S. pasteurii*) is proposed in this paper. The influences of different mixing modes on the cementation effect are investigated so as to improve the cementation effect.

**Comment**

2. There should be a space between number and units. For example, 70mm should be written as 70 mm.

**Response**

Thanks for the comment. This problem has been explained in detail in the manuscript.

The mixture was put into a steel mold (diameter 50 mm, height 100 mm, as shown in Fig.4a) for shaping and sample preparation. In order to avoid loss of tailings particles during the contact process between the cementing solution and the tailing specimen, the specimen was wrapped with 1 mm thick geotextile and laterally confined by a 3D printed mold (as shown in Fig.4b), so as to prevent specimen deformation during immersion curing.

**Comment**

3. I wonder, what is the new finding in the manuscript, comparing to the published article? If it is optimization, that has already been published in previous works. Authors should highlight this in the latter part of the introduction section.

**Response**

Thanks for the comment. In contrast to published articles, the novel findings in this manuscript are the use of a mixture of facultative anaerobes and aerobic bacteria to cement tailings during soak curing. We have emphasized this in the second half of the Introduction section.

To sum up, a tailings cementation method by mixed facultative anaerobes (*Castellaniella denitrificans* or *C. denitrificans* ) and *Sporosarcina pasteurii* (*S. pasteurii*) is proposed in this paper. The influences of different mixing modes on the cementation effect are investigated so as to improve the cementation effect. Based on the research in this paper, it is found that in the process of soaking and curing, the cementation effect is better when facultative anaerobic bacteria and aerobic bacteria are used to act together on the tailings.

**Comment**

4. When writing OD_600_, 600 should be subscripted.

**Response**

Thanks for the comment. This problem has been explained in detail in the manuscript.

When OD_600_ of the *S. pasteurii* solution was 1.6, the cementation effect was no longer enhanced rapidly with increasing bacterial solution concentration.

**Comment**

5. What is the advantage in immersion curing, compared to the spraying/ injection/ percolation? How the proposed method is suitable for tailing applications? The discussion should be expanded in a way to answer these questions.

**Response**

Thanks for the comment. Compared with spraying/ injection/ percolation, immersion curing has the advantages of sufficient cement solution, simple test method and easier practical application. We have expanded the discussion in the text in a way that answers these questions. The original manuscript has been revised accordingly.

Finally, the upper and lower ends of the specimen were wrapped by geotextile (Fig.4c). 30 L cementing solution was injected into the plexiglass box, and the power supply of the magnetic stirrer and the thermostatic heating rod was connected. When a preset constant temperature was reached, the tailings specimen was put into the cementing solution and the air pump was turned on. After being immersed and cured under a constant temperature in an aerobic environment for 28 d, the specimen was taken out and dried for demolding. Tests were then conducted on the tailings specimen for analysis of the cementation effect. According to previous studies, the spray repair method based on MICP technology is limited to repairing and coating the surface cracks of the specimen. The injection and percolation repair methods based on MICP technology are limited to the application in earthwork construction, and the process is relatively complicated and the cost is high. If it is applied to the curing of tailings, the cost is too high and the economic benefit is poor. In this paper, the tailings are cured by immersion curing. Compared with spraying, injection and percolation repair methods, immersion curing has the advantages of good effect, simple technology and easy practical application.

**Comment**

6. Similar to Ordinary Portland cement, the use of MICP also has adverse effect related to the ammonia emission/ release. The authors should briefly discuss the negative side of MICP technique, and should propose the possible methods to overcome this issue. Several recently published works confirmed that the ammonium by-products can be managed by struvite precipitation (https://doi.org/10.1007/s13762-021-03138-z), zeolite use (https://doi.org/10.1520/GTJ20170353), and etc. The authors are recommended to refer those papers and expand the discussion.

**Response**

Thanks for the comment. This problem has been explained in detail in the manuscript. We have discussed in the revised manuscript the issue and possible solutions for ammonia emissions from MICP technology.

As can be seen from Fig.9, a large amount of CaCO_3_ is formed and closely bound in the cemented specimen, which greatly enhances the specimen strength. Therefore, when OD_600_ exceeds 1.6, further increase of bacterial solution concentration cannot improve the concentration effect of microbes on the tailings. This part of the research proves that the MICP technology based on *S. pasteurii* can solidify the tailings into specimens with certain strength under the condition of immersion curing. It is confirmed that *S. pasteurii* has the potential to replace Portland cement as the cementing material of tailings. However, the urea hydrolysis of *S. pasteurii* will increase the emission of ammonia, which will also have a certain impact on the environment. In the follow-up, it can be considered to solve this problem by adjusting the concentration of reactants and adding ammonia gas absorbent.

**Comment**

7. I still confused about one thing. Can this be practical to implement MICP to tailing? In the MICP, the bacteria as well as resources should reach the depths to got treated at those levels. However, the tailing materials are more likely to be very fine (in the range of nano scale). Will it be possible to penetrate the bacteria? Moreover, tailing used to have organic content. A recent study showed only a limited improvement is possible in fine and organic soil materials. Refer the following paper (https://doi.org/10.3389/fenvs.2021.690376) and enhance the discussion, which helps to improve the manuscript standard.

**Response**

Thanks for the comment. We believe that MICP technology is feasible when curing tailings. When the MICP technology is used to solidify the tailings, the microbial bacteria solution is first mixed with the tailings, so the bacteria will exist between the tailings particles. In addition, when the tailings particles are very fine, it can be considered to mix the tailings particles with a larger particle size to play the role of aggregate in the backfill, and bacteria and cementing solutions can exist in the gaps between the tailings particles and the tailings. Different components of the tailings will definitely have a certain impact on the MICP technology. We should select suitable bacteria for the different components of the tailings to solidify them.

**Comment**

8. Regarding the concentration of cementation solutions, 0.25 mol/L, 0.50 mol/L, 0.75 mol/L, 1.00 mol/L and 1.25 mol/L, what is the demerit in using 1 mol/L or 1.25 mol/L? Few previous works recommend the use of 1 mol/L. Scientific explanation may be needed.

**Response**

Thanks for the suggestion. In the experiment process, it was found that when the concentration of the cementing solution exceeds 0.75 mol/L, the cementing effect of microorganisms on tailings will decrease with the increase of the concentration of the cementing solution. It can be seen through analysis that the excessive urea in the solution will affect the growth and reproduction of microorganisms, and the large amount of ammonia by-products can hinder the progress of urea hydrolysis.

**Comment**

9. In some places, 3 in CaCO_3_ is not subscripted. Revision needed throughout.

**Response**

Thanks for the suggestion. This problem has been explained in detail in the manuscript.

Hence, the cementation effect is poor. It can be seen from Fig.15, when the proportion of small or medium-size particles is too high, little CaCO_3_ is formed in the pores between small particles and hence cannot bind fine particles. Therefore, the strength of the cemented tailings which was ground for 90 min is relatively low. Among the tailings specimens with different particle size distributions (except the original tailings) , the cementation effect for the specimen with a mass ratio of 1:1:1:1 is the best, as the existence of larger particles in the specimen with a mass ratio of 1:1:1:1 provides support and enough space for the survival and growth of *S. pasteurii*. Furthermore, the small particles act as fine aggregates between pores. Hence, the coarse and fine particles can still be cemented when the CaCO_3_ crystals formed by the MICP process are small and the cementation effect is good.

**Comment**

10. Is this the first article using *Castellaniella denitrificans*?

**Response**

This article is the first article to use *Castellaniella denitrificans* gum ending sand. Previous studies on the MICP technology for *Castellaniella denitrificans* were used, but the tailings were not cemented. (E.g: Paassen LAV, Daza CM, Staal M, Sorokin DY, Zon WVD, Loosdrecht MCMV. Potential soil reinforcement by biological denitrification. Ecol Eng. 2010;36(2):168-75. http://doi.org/10.1016/j.ecoleng.2009.03.026)

The valuable comments by the two reviewers have greatly helped us to better summarize the results and present the conclusions more clearly and intuitively. The revised manuscript is expected to provide more valuable information to the readers. We sincerely hope that the revised manuscript will be assessed suitable for publication in your esteemed journal. If further information is needed, please do not hesitate to contact us.

Once again, thank you very much for your comments and suggestions.

Best regards,

Changyu Jin
